# Early-Life Exposure to Acid-Suppressive Therapy and the Development of Celiac Disease Autoimmunity

**DOI:** 10.1001/jamanetworkopen.2025.3376

**Published:** 2025-04-04

**Authors:** Tomer Achler, Tal Patalon, Sivan Gazit, Shlomi Cohen, Ron Shaoul, Amir Ben-Tov

**Affiliations:** 1 School of Medicine, Tel Aviv University Faculty of Medical and Health Sciences, Tel Aviv, Israel; 2 Maccabi Research & Innovation Center, Maccabi Healthcare Services, Tel Aviv, Israel; 3 Pediatric Gastroenterology Institute, Dana-Dwek Children’s Hospital, Tel-Aviv Sourasky Medical Center, Tel Aviv, Israel; 4 Pediatric Gastroenterology & Nutrition Institute, Ruth Children’s Hospital of Haifa, Rambam Medical Center, Faculty of Medicine, Technion, Haifa, Israel

## Abstract

**Importance:**

Early-life use of acid-suppressive therapy has increased over the past 2 decades. Although these medications are widely used, recent studies showed an association between early-life use of acid-suppressive therapy and various long-term outcomes, including celiac disease.

**Objective:**

To assess the association between early-life use of acid-suppressive therapy and the risk of celiac disease autoimmunity using 2 observational approaches on a large population-based database.

**Design, Setting, and Participants:**

The cohort study took place in Israel using Maccabi Healthcare Services data. The data were collected on December 8, 2023, and were initially analyzed from January to May 2024. Analysis of the data continued during the revision rounds that took place from October 2024 to February 2025. Children born between January 1, 2005, and December 31, 2020, were included, grouped based on their exposure to acid-suppressive therapy within the first 6 months after birth and subsequently followed up for outcome development until the age of 10 years or December 8, 2023. A retrospective matched cohort design (N = 79 820) and retrospective matched test-negative case-control design (n = 24 684), including only the population tested for celiac disease autoimmunity, were used separately and compared.

**Exposure:**

Prescription purchase of acid-suppressive therapy, either proton-pump inhibitors or histamine-2 receptor antagonists, during the first 6 months of life.

**Main Outcomes and Measures:**

Celiac disease autoimmunity was defined as a positive anti–transglutaminase 2 enzyme-linked immunosorbent assay test result according to the thresholds of the commercial kits used. Time to first positive result for celiac disease autoimmunity was defined as the outcome in the cohort design, and acid-suppressive therapy use was defined as the outcome in the test-negative design.

**Results:**

The cohort design included 79 820 children (41 319 boys with no acid-suppressive therapy use [51.8%]; median birth year, 2015 [IQR, 2011-2018]), of whom 19 955 (25.0%) used acid-suppressive therapy. The rate of celiac disease autoimmunity was significantly higher among children using acid-suppressive therapy than among those not using acid-suppressive therapy (1.6% [310 of 19 955] vs 1.0% [610 of 59 865]; *P* < .001). The adjusted hazard ratio of acid-suppressive therapy use for development of celiac disease autoimmunity was 1.52 (95% CI, 1.33-1.74). In the test-negative case-control design, a total of 24 684 children were included (62.2% girls; median birth year, 2012 [IQR, 2009-2016]), of whom 6176 (25.0%) were celiac disease autoimmunity positive. The rate of acid-suppressive therapy users among those who tested positive for celiac disease autoimmunity was not significant compared with those who tested negative (5.0% [309 of 6176] vs 4.6% [858 of 18 508]; *P* = .25). The adjusted odds ratio of a positive celiac disease autoimmunity test for acid-suppressive therapy use was 1.07 (95% CI, 0.94-1.23), which was nonsignificant compared with the population that tested negative.

**Conclusions and Relevance:**

This retrospective study included both cohort and test-negative case-control designs. In the cohort design, acid-suppressive therapy was significantly associated with celiac disease autoimmunity. In the test-negative case-control design, this association was not significant. These results suggest a residual confounding by health care utilization in cohort designs studying celiac disease and suggest a noncausal association between acid-suppressive therapy and celiac disease autoimmunity.

## Key Points

**Question:**

Is acid-suppressive therapy during early life associated with the development of celiac disease?

**Findings:**

This study included both a cohort design and a test-negative case-control design. Acid-suppressive therapy was associated with celiac disease autoimmunity only in the cohort study of 79 820 children and not in the test-negative case-control study of 24 684 children.

**Meaning:**

This study suggests that the association between early-life use of acid-suppressive therapy and development of celiac disease is suspected to be confounded by health care utilization behavior.

## Teaser

This study uses both a cohort design comprising 79 820 children and a test-negative case-control design comprising 24 684 children to assess the association between early-life use of acid-suppressive therapy and the risk of celiac disease autoimmunity.

## Introduction

Use of acid-suppressive therapy, including proton-pump inhibitors (PPIs) and histamine-2 receptor antagonists (H2RAs), during early life has been increasing worldwide in recent years. Observational studies found an association between the early-life use of these medications and long-term adverse effects, including fractures^1,2^ and celiac disease.^3^

Celiac disease is an immune-mediated enteropathy. The prevalence and incidence of celiac disease have increased in most countries during the past decades,^4^ both for seropositive celiac disease, defined as celiac disease autoimmunity, and for biopsy-confirmed celiac disease. Transglutaminase 2 (TG2), an enzyme secreted into the extracellular matrix during inflammation, plays an important role in the pathophysiology of celiac disease by promoting the creation of antigliadin antibodies. Anti-TG2 antibodies are also produced during this inflammatory process and are used in celiac disease serologic tests.^5^ One of the first steps in the cascade of events in celiac disease development is the appearance of celiac disease–specific antibodies^5,6^; almost all children with celiac disease have celiac disease autoimmunity.^7^ The accuracy of serologic tests for the diagnosis of celiac disease is well recognized and contributed to the “no-biopsy” approach to diagnosis in cases with high rates of positive serologic findings among children.^8,9^ Few mechanisms have been suggested to link acid-suppressive therapy and celiac disease, including incomplete protein degradation and microbiome alternations.^3^

Although observational studies play an important role in medical knowledge by helping to assess the clinical effectiveness of medical interventions and identifying rare or long-term adverse events, they are susceptible to confounding,^10^ and their results should be interpreted with caution.^11^ Most of these studies used matched control cohorts for their analysis. Therefore, we used our robust, large, population-based database of infants exposed to acid-suppressive therapy during early life and children with celiac disease autoimmunity to investigate their association using 2 observational approaches: a matched control design and a test-negative case-control design (TND).

## Methods

### Settings

Two retrospective observational approaches were used and compared: a matched cohort study and a matched TND on the large population-based deidentified database of Maccabi Healthcare Services (MHS). Maccabi Healthcare Services is the second-largest state-mandated health fund in Israel, covering 27.3% of the Israeli population (2.7 million) and with similar sociodemographic characteristics to the Israeli population and a 1% annual turnover rate of its members. The broad coverage of MHS allows this study to represent the entire Israeli population, and the low turnover rate enables a long follow-up period with low dropout. The database contains a comprehensive medical history, including inpatient and outpatient procedures, laboratory tests, and prescription medication purchase history. The laboratory tests are analyzed in a central mega-laboratory. The data were institutional level, and no external data linkage was done. The data were collected on December 8, 2023. Ethical approval was obtained from the MHS Ethics Committee, which waived consent because the data were deidentified. This case-control cohort study followed the Strengthening the Reporting of Observational Studies in Epidemiology (STROBE) reporting guideline.^11^

### Study Population

**Figure 1.**
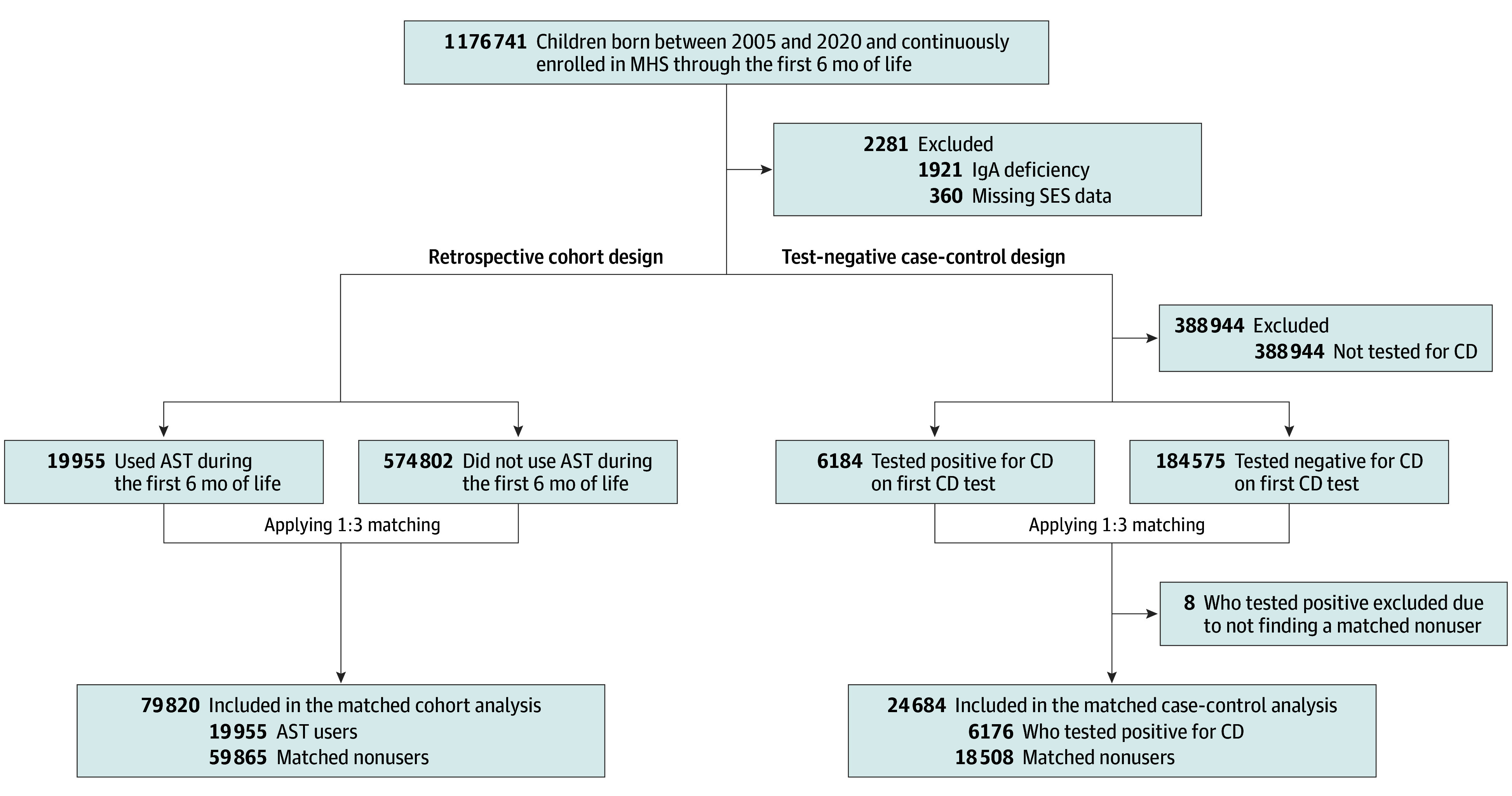
Flow Diagram Flow diagram showing number of participants at each stage. AST indicates acid-suppressive therapy; CD, celiac disease; IgA, immunoglobulin A; MHS, Maccabi Healthcare Services; and SES, socioeconomic status.

All children born between January 1, 2005, and December 31, 2020, who joined MHS during their first week of life and did not receive a diagnosis of immunoglobulin A (IgA) deficiency throughout the study period were eligible to be included in the study. Children with IgA deficiency were excluded because this rare condition (0.3% of children in our base population, as shown in Figure 1) required different less sensitive serologic tests to diagnose celiac disease. In the cohort analysis, children who used acid-suppressive therapy during early life were matched with up to 3 nonusers. The participants were followed up until the earliest of reaching the age of 10 years, leaving MHS, or reaching an outcome. Ending the follow-up at 10 years of age was chosen to balance between capturing the peak incidence of celiac disease autoimmunity from 0 to 10 years of age^12^ and maintaining temporal proximity between the exposure and the outcome. Two outcomes were tested separately: receipt of a diagnosis of celiac disease autoimmunity or testing for celiac disease autoimmunity. In the TND, children who were tested for celiac disease autoimmunity until the age of 10 years were included in the study. Each child with a positive result for celiac disease autoimmunity was matched with up to 3 children with a negative result for celiac disease autoimmunity, and early-life acid-suppressive therapy use was evaluated.

### Variables

Early-life acid-suppressive therapy use was defined as a purchase of either a PPI (omeprazole) or H2RA (ranitidine) for 1 month of use during the first 6 months of life. Using acid-suppressive therapy more than once was defined as more than 1 purchase of acid-suppressive therapy. A diagnosis of celiac disease autoimmunity was based on a positive result of an anti-TG2 enzyme-linked immunosorbent assay (ELISA) test. The MHS central laboratory used 2 TG2-IgA ELISA commercial kits during the study period: Celikey (ThermoFisher) from 2005 to 2011 and Elia (ThermoFisher) from 2012 onward. The positive thresholds, based on the manufacturer’s instructions, were 5 U/mL for the Celikey test and 7 U/mL for the ELISA test. For positive results, the maximal upper limit of normal value was calculated based on the first 10 tests by dividing the numerical result by the corresponding threshold. The following variables were used for matching in both methods: sex, birth year, and socioeconomic status (SES). These variables were chosen based on their clinical relevance to both acid-suppressive therapy and celiac disease autoimmunity. The SES variable was based on the Israeli Central Bureau of Statistics^13^ with a scale of 1 to 10 (where 1 indicates low status and 10 indicates high status). The variables of test year, age when tested, and test count (truncated by 10) were additionally used in the TND for matching. These variables were calculated for the celiac disease autoimmunity–positive population as the first celiac disease autoimmunity test and as the latest test (before the end of the follow-up) for the celiac disease autoimmunity–negative population.

### Outcomes

The primary outcome of the cohort analysis was a positive serologic, test-based, celiac disease autoimmunity diagnosis between 6 months of life and the age of 10 years. Being tested for celiac disease autoimmunity was used as an additional outcome.

### Statistical Analysis

The data were initially analyzed from January to May 2024. Analysis of the data continued during the revision rounds that took place from October 2024 to February 2025. A description of the children, broken into the different study groups (acid-suppressive therapy users vs nonusers and celiac disease autoimmunity test positive vs negative), was conducted for each study design. Continuous variables were described using median (IQR) values, and categorical variables were described using absolute values and percentages. The matching in both methods was done using generalized linear models for calculating distances, with variable-specific calipers and exact matching for selected variables. Matching was done using nearest neighbor algorithms from the MatchIt R package, version 4.5.5 (R Project for Statistical Computing), while allowing up to 3 nonexposed children or those who tested negative for celiac disease autoimmunity to be matched with 1 exposed child or child who tested positive for celiac disease autoimmunity. The following variable-specific calipers (maximum allowable differences) were applied for matching: a caliper of 1 unit was used for SES in both designs, a caliper of 4 months was used in the TND for celiac disease autoimmunity test age, and a caliper of 2 tests was used in the TND for the number of celiac disease autoimmunity tests. The continuous variables of birth year and celiac disease autoimmunity test year were matched exactly with no calipers. The quality of the match was evaluated using propensity score density plots and standardized mean differences while allowing values lower than 0.1 for matching covariates.

In the cohort method, Kaplan-Meier curves with a log-rank test were used as a univariate analysis, and the Cox proportional hazards regression model was used to calculate the adjusted hazard ratio (AHR) associated with acid-suppressive therapy use, comparing children with 2 or more purchases vs those with 0 to 1 purchase and using celiac disease autoimmunity test (rather than positive celiac disease autoimmunity test) as the outcome. The Cox proportional hazards regression model is a survival analysis model that considers the survival time contributed by each participant and handles right censoring of participants who did not complete the follow-up period. The following models were used as a sensitivity analysis: a Cox proportional hazards regression model using unmatched data and a Cox proportional hazards regression model using the unmatched data where the celiac disease autoimmunity outcome was defined as 10 times the upper limit of normal. The Cox proportional hazards regression models were adjusted for the variables used in the matching process and were stratified for any variable for which the Schoenfeld residuals were significantly correlated with the time to meet the proportional hazards assumption. In the TND, a logistic regression, adjusted for the variables used for matching, was used to calculate the adjusted odds ratio (AOR) of acid-suppressive therapy among groups. Missing values in categorical variables were grouped into a “missing” category, while children with missing SES data were excluded from the analysis, resulting in no missing values for this continuous variable. All *P* values were from 2-sided tests, and results were deemed statistically significant at *P* < .05. The analysis was conducted using R, version 4.1.0 (R Project for Statistical Computing).

## Results

A total of 597 038 children were born between 2005 and 2020 and continuously enrolled in MHS through the first 6 months of life. Of the total population, 2281 were excluded due to IgA deficiency (n = 1921) or missing SES (n = 360). In the cohort design, a total of 79 820 matched children were included, of whom 53.6% were boys, 46.4% were girls, the median birth year was 2015 (IQR, 2011-2018]), and 19 955 (25.0%) were using acid-suppressive therapy (Figure 1; Table 1). In the TND, a total of 24 684 children were included, of whom 37.8% were boys, 62.2% were girls, the median birth year was 2012 (IQR, 2009-2016), and 6176 (25.0%) were celiac disease autoimmunity positive (Figure 1; Table 2). Overall, the celiac disease autoimmunity rate was 1.0%; the overall celiac disease positivity rate was 3.2% (6184 of 190 759), and the median age at celiac disease diagnosis in the cohort design and in the TND were 4.7 years (IQR, 3.3-6.2 years) and 4.8 years (IQR, 3.3-6.6 years), respectively. In both designs, the standardized mean differences after matching were smaller than 0.1 (Table 1 and Table 2).

**Table 1.** Baseline Characteristics of Acid-Suppressive Therapy Users vs Nonusers in the Cohort Analysis, Before and After Matching

Characteristic	Before matching			After matching		
	No acid-suppressive therapy use (n = 574 802)	Acid-suppressive therapy use (n = 19 955)	SMD^a^	No acid-suppressive therapy use (n = 59 865)	Acid-suppressive therapy use (n = 19 955)	SMD^a^
Sex, No. (%)						
Male^b^	294 553 (51.2)	10 687 (53.6)	0.05	30 632 (53.6)	10 687 (53.6)	<0.001
Female	280 249 (48.8)	9268 (46.4)	0.05	26 524 (46.4)	9268 (46.4)	<0.001
Birth year, median (IQR)^b^	2013 (2009-2017)	2015 (2011-2018)	0.34	2015 (2011-2018)	2015 (2011-2018)	<0.001
SES, median (IQR)^b^^,^^c^	6.0 (4.0-8.0)	7.0 (5.0-9.0)	0.25	7.0 (5.0-9.0)	7.0 (5.0-9.0)	<0.001
Celiac disease autoimmunity positive, No. (%)	5874 (1.0)	310 (1.6)	0.05	610 (1.0)	310 (1.6)	0.05
Maximal celiac disease autoimmunity test ULN category, No. (% of celiac disease autoimmunity positive)^d^						
1-3	1500 (25.5)	90 (29.0)	0.10	149 (24.4)	90 (29.0)	0.13
3-7	799 (13.6)	45 (14.5)		87 (14.3)	45 (14.5)	
7-10	315 (5.4)	17 (5.5)		27 (4.4)	17 (5.5)	
>10	3260 (55.5)	158 (51.0)		347 (56.9)	158 (51.0)	
Tested for celiac disease, No. (%)	181 801 (31.6)	8958 (44.9)	0.28	19 216 (32.1)	8958 (44.9)	0.27
Celiac disease autoimmunity diagnosis age, median (IQR), y	4.8 (3.4-6.7)	4.6 (3.3-6.1)	0.03	4.7 (3.4-6.3)	4.6 (3.3-6.1)	0.13

Abbreviations: SES, socioeconomic status; SMD, standardized mean difference; ULN, upper limit of normal.

^a^ SMD is the difference between the groups’ means divided by the pooled SD (<0.1 is considered a minor difference).

^b^ Covariates used for matching.

^c^ Residential area SES by the Central Bureau of Statistics census data; range, 1 to 10 (where 1 indicates low status and 10 indicates high status).

^d^ Greater than the lower boundary of the category and greater than or equal to the higher boundary of the category.

**Table 2.** Baseline Characteristics Per Celiac Disease Test Status in the Test-Negative Case-Control Analysis, Before and After Matching

Characteristic	Before matching				After matching		
	Not tested (n = 403 998)	Celiac disease negative (n = 184 575)	Celiac disease positive (n = 6184)	SMD^a^	Celiac disease negative (n = 18 508)	Celiac disease positive (n = 6176)	SMD^a^
Sex, No. (%)							
Male^b^	208 337 (51.6)	94 563 (51.3)	2340 (37.8)	0.19	7005 (37.8)	2337 (37.8)	<0.001
Female	195 661 (48.4)	90 012 (48.7)	3844 (62.2)	0.19	11 503 (62.2)	3839 (62.2)	<0.001
Birth year, median (IQR)^b^	2013 (2009-2017)	2012 (2009-2016)	2012 (2009-2016)	0.11	2012 (2009-2016)	2012 (2009-2016)	0.001
SES, median (IQR)^b^^,^^c^	6.0 (3.0-8.0)	7.0 (5.0-9.0)	7.0 (5.0-9.0)	0.24	7.0 (5.0-9.0)	7.0 (5.0-9.0)	0.01
Celiac disease test year, median (IQR)^b^	NA	2018 (2015-2021)	2018 (2014-2021)	0.07	2018 (2015-2021)	2018 (2015-2021)	0.001
Celiac disease test age, median (IQR), y^b^	NA	5.5 (3.2-7.8)	4.8 (3.4-6.6)	0.17	4.9 (3.4-6.6)	4.8 (3.4-6.6)	<0.001
No. of celiac disease tests, median (IQR)^b^^,^^d^	NA	1.0 (1.0-2.0)	1.0 (1.0-2.0)	0.16	1.00 (1.0-2.0)	1.0 (1.0-2.0)	0.03
Acid-suppressive therapy use, No. (%)	10 997 (2.7)	8648 (4.7)	310 (5.0)	0.08	858 (4.6)	309 (5.0)	0.02

Abbreviations: NA, not applicable; SES, socioeconomic status; SMD, standardized mean difference.

^a^ SMD is the difference between the groups’ means divided by the pooled SD (<0.1 is considered a minor difference).

^b^ Covariates used for matching.

^c^ Residential area SES by the Central Bureau of Statistics census data; range, 1 to 10 (where 1 indicates low status and 10 indicates high status).

^d^ The number of positive tests for those who tested positive and the number of latest tests for those who tested negative.

### Matched Cohort Analysis

**Figure 2.**
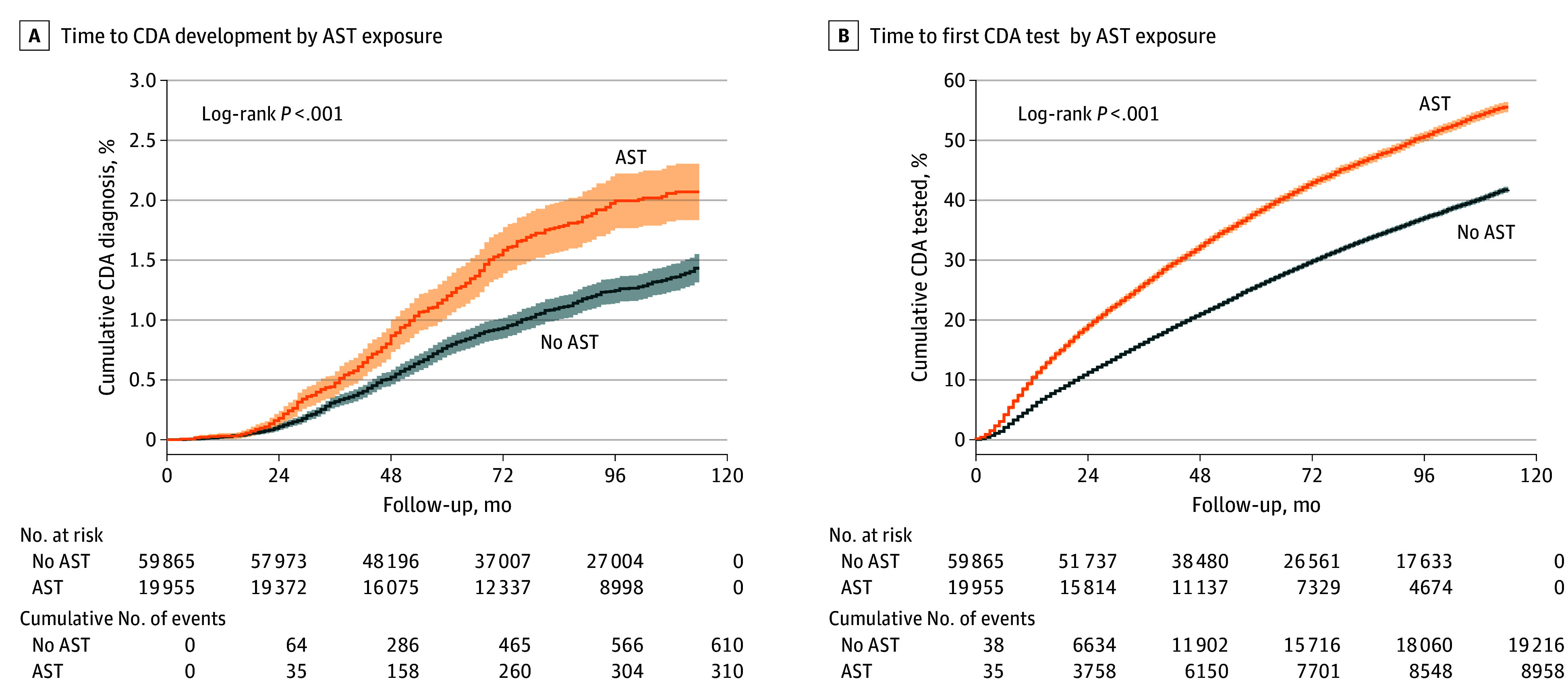
Kaplan-Meier Curves for Celiac Disease Autoimmunity (CDA) and CDA Test, Stratified by Acid-Suppressive Therapy (AST) Exposure A, Time to CDA development stratified by AST exposure. B, Time to first CDA test performance stratified by AST exposure.

Prior to matching (594 757 children, of whom 6184 were celiac disease positive), the unadjusted hazard ratio of using acid-suppressive therapy for celiac disease was 1.64 (95% CI, 1.46-1.84). Of 19 955 children using acid-suppressive therapy, 310 (1.6%) received a diagnosis of celiac disease autoimmunity, significantly higher than the 5874 of 574 802 nonusers (1.0%) who received a diagnosis of celiac disease autoimmunity (*P* < .001). After matching, the celiac disease autoimmunity rates did not differ significantly from prematching rates of 1.6% (310 of 19 955; *P* > .99) among acid-suppressive therapy users and 1.0% (610 of 59 865; *P* = .96) among nonusers. In the matched population, the median follow-up time was 88 months (IQR, 55-113 months). Kaplan-Meier curves with a log-rank test showed a significant association between acid-suppressive therapy and celiac disease autoimmunity (χ^2^ = 36.9; *P* < .001) (Figure 2A). The AHR of acid-suppressive therapy use during the first 6 months of life for the development of celiac disease autoimmunity was 1.52 (95% CI, 1.33-1.74). Using acid-suppressive therapy for more than 1 month was associated with celiac disease autoimmunity (AHR, 1.65; 95% CI, 1.34-2.03). The AHR of PPI use for celiac disease autoimmunity was 1.57 (95% CI, 1.25-1.97), and the AHR of H2RA use was 1.51 (95% CI, 1.29-1.76).

Acid-suppressive therapy users had a significantly higher celiac disease autoimmunity test rate than nonusers (44.9% [8958 of 19 955] vs 32.1% [19 216 of 59 865; *P* < .001) (Table 1). The median follow-up time until celiac disease autoimmunity test was 62 months (IQR, 34-103 months). Univariate testing showed that acid-suppressive therapy was associated with performance of celiac disease autoimmunity serologic testing (AHR, 1.57; 95% CI, 1.53-1.61; *P* < .001) (Figure 2B). Using acid-suppressive therapy more than once was associated with celiac disease autoimmunity testing (AHR, 1.74; 95% CI, 1.68-1.82). Use of PPIs was associated with celiac disease autoimmunity testing (AHR, 1.63; 95% CI, 1.56-1.70), as was use of H2RAs (AHR, 1.55; 95% CI, 1.51-1.60). The models were stratified for variables that were significantly associated with time based on Schoenfeld residuals (eTable 1 in Supplement 1). The Schoenfeld residuals tests were significant (χ^2^ = 70.6; *P* < .001) for the acid-suppressive therapy covariates in the models when the outcome was celiac disease autoimmunity testing (eTable 1 in Supplement 1).

### Test-Negative Case-Control Analysis

The rate of acid-suppressive therapy use among children who tested positive for celiac disease autoimmunity serologic findings was 5.0% (309 of 6176), nonsignificantly higher than the rate among children who tested negative (858 of 18 508 [4.6%]; *P* = .25) (Table 2). The AOR of a positive celiac disease autoimmunity test result for acid-suppressive therapy was 1.07 (95% CI, 0.94-1.23), which was nonsignificant compared with the population with a negative test result. Among children positive for celiac disease autoimmunity, girls were more likely to have a maximal celiac disease autoimmunity test result greater than 10 times the upper limit of normal than a result 10 times or less than the upper limit of normal (63.3% [2163 of 3415] vs 60.7% [1676 of 2761]; *P* = .04) (eTable 2 in Supplement 1). The acid-suppressive therapy use rate was not significantly different between children with a maximal celiac disease autoimmunity test result greater than 10 times the upper limit of normal than those with a result 10 times or less than the upper limit of normal (4.6% [158 of 3415] vs 5.5% [151 of 2761]; *P* = .15). The AOR of celiac disease autoimmunity associated with using acid-suppressive therapy more than once was not significant (1.09 [95% CI, 0.91-1.36]), and the AOR of celiac disease autoimmunity was 1.00 (95% CI, 0.78-1.27) for PPI use and 1.11 (95% CI, 0.78-1.29) for H2RA use.

### Sensitivity Analyses

Acid-suppressive therapy was associated with celiac disease autoimmunity in an unmatched multivariate Cox proportional hazards regression model (AHR, 1.47; 95% CI, 1.31-1.65) (eTable 3 in Supplement 1). An additional sensitivity analysis restricted to children positive for celiac disease autoimmunity showed a significant association between acid-suppressive therapy and high positive serology rates of more than 10 times the upper limit of normal (eTable 4 in Supplement 1).

## Discussion

This population-based retrospective case-control cohort study showed mismatched results in the association between acid-suppressive therapy and celiac disease autoimmunity when using different study methods. In the cohort design, acid-suppressive therapy was significantly associated with celiac disease autoimmunity (AHR, 1.52; 95% CI, 1.33-1.74), with a further increased AHR of 1.65 (95% CI, 1.34-2.03) when acid-suppressive therapy was used for more than 1 month during the first 6 months of life. The AHR of acid-suppressive therapy for being tested for celiac disease autoimmunity was also significant (AHR, 1.57; 95% CI, 1.53-1.61) and showed a further increased AHR for those who used acid-suppressive therapy more than once (AHR, 1.74; 95% CI, 1.68-1.82). In the TND, celiac disease autoimmunity was not associated with either any early-life acid-suppressive therapy use or second use onward.

The cohort design showed an association between use of acid-suppressive therapy and celiac disease autoimmunity and a stronger association with prolonged use, which may imply a dose-response relationship, one of the Bradford Hill criteria for causality.^14^ However, the similarity between the HRs of acid-suppressive therapy for positive celiac disease autoimmunity and those for being tested for celiac disease autoimmunity (especially given our prematching clinical results of a 3.2% positivity rate) suggests a possible noncausal association and encouraged us to seek an alternative study design that considers the chances of being tested. Using a TND allowed for neutralizing the factors influencing celiac disease autoimmunity test performance by matching children with positive test results with those with negative test results and using the number of tests performed, the age of the child, and the year the test took place for matching.

The TND became popular during the past decade to calculate vaccine effectiveness against various pathogens.^15^ It was used in the latest COVID-19 pandemic to calculate vaccine effectiveness^16^ or waning effectiveness of vaccination.^17^ The TND allows for control for health care accessibility and health-seeking behavior as the study population is restricted to patients who had access to health care services.^18^ It was found valid compared with a randomized clinical trial^19^ and with a cohort design with explicit target trial emulation.^20^ The eFigure in Supplement 1 shows the directed acyclic graph of our hypothesized association between acid-suppressive therapy and celiac disease autoimmunity. The TND conditioned the study on the tested population and blocked the backdoor pathway between acid-suppressive therapy and celiac disease autoimmunity. Because this pathway was not blocked in the cohort analysis, the significant association showed that using the cohort design might have reflected this pathway. Although matching and adjustment for possible confounders decreased the AHR from 1.64 in the unmatched population to 1.52 in the matched population of the cohort design, residual confounding associated with the likelihood of being tested for celiac disease autoimmunity remained, as this pathway could not be fully controlled for in the cohort approach. To our knowledge, this is the first study using TND in the domain of celiac disease.

Boechler et al^3^ conducted a retrospective cohort study and found a significant association between acid-suppressive therapy and celiac disease, with an AHR of 1.94 for H2RAs and an AHR of 2.23 for PPIs comparable with the significant AHR of 1.51 for H2RAs and 1.57 for PPIs found in our study. Our cohort design and the study by Boechler et al^3^ share a similar study design and exposure definition. Our study differs by its higher celiac disease rate (1.0% vs 0.2%) and use of serology-based outcomes rather than *International Classification of Diseases, Ninth Revision* (*ICD-9*)–based outcomes. Boechler et al^3^ suggested 4 causal mechanisms for this association: protein degradation, mucosal permeability, microbiome changes, and immune reactivity. Although these mechanisms seem plausible and supported by basic science studies, we suggest an alternative explanation in which the use of acid-suppressive therapy is merely a sign of increased health awareness of parents that is reflected in the higher rate of tests performed in this population. In contrast to *ICD-9* code–based studies, the advantages of controlling for whether the test for celiac serology was performed allowed us to differentiate the association that might have a causal biologic mechanism with those associated with behavior.

Both celiac disease autoimmunity^12^ and acid-suppressive therapy use^21^ rates increased in recent years in Israel. Celiac disease and acid-suppressive therapy use were also associated independently with higher SES.^21,22^ As was suggested in a recent study^1^ that examined the association between acid-suppressive therapy and fractures, the parent’s behavior could confound this association, as health-seeking parents may drive health care professionals to prescribe acid-suppressive therapy for gastroesophageal reflux–like symptoms during infancy and to test for celiac disease autoimmunity during childhood more frequently or for milder gastroesophageal symptoms.

### Strengths and Limitations

This study has some strengths, including the use of the population-based database with low turnover, high coverage of variables, and the use of serologic tests to define the outcome rather than using a celiac disease diagnosis from the medical record. Unlabeled purchases of acid-suppressive therapy are rare because a membership card is required when purchasing prescribed medications in Israel.

This study also has some limitations. It was limited to an association with celiac disease autoimmunity and not with biopsy-proven celiac disease. The use of celiac disease autoimmunity as an outcome is now widely used in celiac disease epidemiologic studies, mainly since 2012 when the European Paediatric Society for Gastroenterology, Hepatology, and Nutrition updated its guidelines to include the possibility of diagnosing celiac disease based on high serology results^23^; this approach was reaffirmed in the 2020 guidelines^9^ and was adopted recently by the American College of Gastroenterology.^8^ Additional limitations of this study are its retrospective nature, missing information about the reasons why the celiac disease tests were performed, and lack of behavior-related covariates that could have shed light on the nature of the association and established the study hypothesis.

## Conclusions

This retrospective case-control cohort study included both a cohort design and TND. The cohort design comprised 79 820 children and showed an association between early-life use of acid-suppressive therapy and celiac disease autoimmunity diagnosis and higher chances to be tested for celiac disease. In the TND, which included 24 684 children, this association was not found. With the increasing rates of celiac disease worldwide, there is increasing interest in its causes^24^; the search for causes, however, should be cautious given the high rate of bias in observational studies. We recommend that studies exploring associations using test results for outcomes in celiac disease should report a test-negative analysis as part of their results.

## Notes

Supplement 1. **eTable 1.** Variables Stratified in Cox Models to Address Non-Proportional Hazards Assumption **eTable 2.** Baseline Characteristics Per Maximal CDA Test ULN in the Case-Control Analysis, Post Matching **eTable 3.** Cox Proportional Hazard Model of Acid-Suppressive Therapy for Celiac Disease Autoimmunity Using Unmatched Data **eTable 4.** Cox Proportional Hazard Model of Acid-Suppressive Therapy for 10 Times Upper Limit Normal Celiac Disease Autoimmunity Using Unmatched Data **eFigure.** Directed Acyclic Graph Showing the Association Between AST and CDA in the Test-Negative Design

Supplement 2.
Data Sharing Statement
