# Supplementary material for: Early-Life Exposure to Acid-Suppressive Therapy and the Development of Celiac Disease Autoimmunity
Source: JAMA Netw Open. 2025 Apr 4;8(4):e253376. doi: 10.1001/jamanetworkopen.2025.3376 (PMC11971667; doi:10.1001/jamanetworkopen.2025.3376)
Supplement: Supplement 1. — eTable 1. Variables Stratified in Cox Models to Address Non-Proportional Hazards Assumption eTable 2. Baseline Characteristics Per Maximal CDA Test ULN in the Case-Control Analysis, Post Matching eTable 3. Cox Proportional Hazard Model of Acid-Suppressive Therapy for Celiac Disease Autoimmunity Using Unmatched Data eTable 4. Cox Proportional Hazard Model of Acid-Suppressive Therapy for 10 Times Upper Limit Normal Celiac Disease Autoimmunity Using Unmatched Data eFigure. Directed Acyclic Graph Showing the Association Between AST and CDA in the Test-Negative Design [file jamanetwopen-e253376-s001.pdf]

Supplementary Online Content

Achler T, Patalon T, Gazit S, Cohen S, Shaoul R, Ben-Tov A. Early-life exposure to acid-suppressive therapy and the development of celiac disease autoimmunity. *JAMA Netw Open.* 2025;8(4):e253376. doi:10.1001/jamanetworkopen.2025.3376

eTable 1. Variables Stratified in Cox Models to Address Non-Proportional Hazards Assumption

eTable 2. Baseline Characteristics Per Maximal CDA Test ULN in the Case-Control Analysis, Post Matching

eTable 3. Cox Proportional Hazard Model of Acid-Suppressive Therapy for Celiac Disease Autoimmunity Using Unmatched Data

eTable 4. Cox Proportional Hazard Model of Acid-Suppressive Therapy for 10 Times Upper Limit Normal Celiac Disease Autoimmunity Using Unmatched Data

eFigure. Directed Acyclic Graph Showing the Association Between AST and CDA in the Test-Negative Design

This supplementary material has been provided by the authors to give readers additional information about their work.

© 2025 Achler T et al. *JAMA Network Open.*

eTable 1. Variables Stratified in Cox Models to Address Non-Proportional Hazards Assumption

Exposure	Outcome	Non-stratified covariates	Stratified covariates	Comment
AST	CDA	Sex, SES	Birth year	
AST level (1,2+)	CDA			
AST type	CDA			
AST	CDA test		Sex, birth year, SES	Schoenfeld residuals were significant (<0.001) for the exposure variable
AST level (1,2+)	CDA test			
AST type	CDA test			

Abbreviations: AST, acid-suppressive therapy; CDA, celiac disease autoimmunity; SES, socioeconomic status.

© 2025 Achler T et al. *JAMA Network Open.*

eTable 2. Baseline Characteristics Per Maximal CDA Test ULN in the Case-Control Analysis, Post Matching

	Maximal ULN $\leq$ 10	Maximal ULN > 10	p
n	2,761	3,415	
Male[a] (%)	1,085 (39.3)	1,252 (36.7)	0.04
Female (%)	1,676 (60.7)	2,163 (63.3)	0.04
Birth year[a], median (IQR)	2013 (2009-2016)	2012 (2009-2016)	0.33
SES[a,b], median (IQR)	7.0 (5.0-9.0)	7.0 (4.0-9.0)	<0.001
AST usage (%)	151 (5.5)	158 (4.6)	0.15

Abbreviations: AST, acid-suppressive therapy; CD, Celiac disease; IQR, interquartile range; SD, standard deviation; SES, socioeconomic status.
[a] Covariates used for propensity score matching
[b] Residential area SES by the central bureau of statistics census data. Range 1-10 (10 indicates high status).
[d] The number of positive test for tested positive and the number of latest test for tested negative.

© 2025 Achler T et al. *JAMA Network Open.*

eTable 3. Cox Proportional Hazard Model of Acid-Suppressive Therapy for Celiac Disease Autoimmunity Using Unmatched Data

	aHR for CD-positive (95% CI)
AST Usage	1.47 (1.31-1.65)
Male	0.58 (0.55-0.61)

Abbreviations: aHR, adjusted hazard-ratio; AST, acid-suppressive therapy; CD, celiac disease.

* Covariates that showed dependency between Schoenfeld residuals and time were used to stratify the model.

© 2025 Achler T et al. *JAMA Network Open.*

eTable 4. Cox Proportional Hazard Model of Acid-Suppressive Therapy for 10 Times Upper Limit Normal Celiac Disease Autoimmunity Using Unmatched Data

	aHR for ten-time ULN CD-positive (95% CI)
AST Usage	1.37 (1.17-1.61)
Male	0.55 (0.51-0.59)
SES[a]	1.06 (1.05-1.08)

Abbreviations: aHR, adjusted hazard-ratio; AST, acid-suppressive therapy; CD, celiac disease; SES, socioeconomic status; ULN, upper limit normal.

* Covariates that showed dependency between Schoenfeld residuals and time were used to stratify the model.

[a] Residential area SES by the central bureau of statistics census data. Range 1-10 (10 indicates high status).

© 2025 Achler T et al. *JAMA Network Open.*

eFigure. Directed Acyclic Graph Showing the Association Between AST and CDA in the Test-Negative Design. The model was adjusted for the baseline covariates (C) and was restricted for tested population (T)

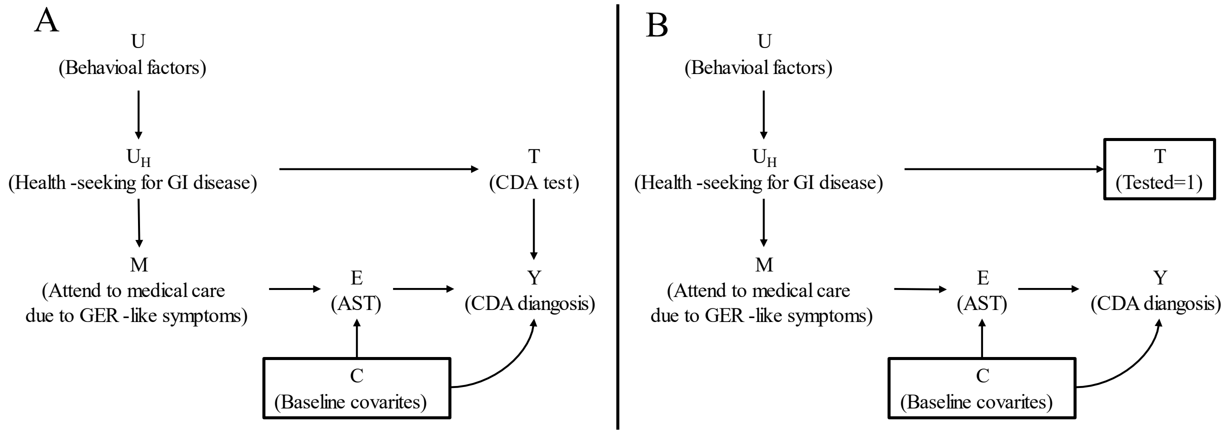

Figure S1. Directed acyclic graph showing the relationship between AST and CDA prior and after applying the test-negative design. U – behavioral factors such as child temperament and mother health-seeking behavior. E – using AST; Y – positive CDA serology test; U_H – health seeking behavior specific for GI diseases; M – attending to doctor due to GER-like symptoms;T – perform a serology test for CDA; C – baseline confounders (sex, birth year, birth order, preterm level, gestational age, and socioeconomic status). Abbreviations: AST – acid-suppressive therapy; CDA – celiac disease autoimmunity; GER – gastroesophageal reflux; GI – gastrointestinal.(A) Before applying the test negative design, a possible backdoor pathway linking E to Y through the common ancestor U_H (B) Conditioning the analysis on the tested population eliminates the backdoor pathway between E and Y.

© 2025 Achler T et al. *JAMA Network Open.*
