# Supplementary material for: Early-Life Exposure to Acid-Suppressive Therapy and the Development of Celiac Disease Autoimmunity
Source: JAMA Netw Open. 2025 Apr 4;8(4):e253376. doi: 10.1001/jamanetworkopen.2025.3376 (PMC11971667; doi:10.1001/jamanetworkopen.2025.3376)
Supplement: Supplement 2. — Data Sharing Statement [file jamanetwopen-e253376-s002.pdf]

Data Sharing Statement

Achler. Early-Life Exposure to Acid-Suppressive Therapy and the Development of Celiac Disease Autoimmunity. *JAMA Netw Open*. Published April 04, 2025.
doi:10.1001/jamanetworkopen.2025.3376

Data
Data available: No

Additional Information
Explanation for why data not available: According to the Israel Ministry of Health regulations, individual-level data cannot be shared openly
